# Video Game–Based Rehabilitation Approach for Individuals Who Have Undergone Upper Limb Amputation: Case-Control Study

**DOI:** 10.2196/17017

**Published:** 2021-02-04

**Authors:** N A Hashim, N A Abd Razak, H Gholizadeh, N A Abu Osman

**Affiliations:** 1 Department of Biomedical Engineering Faculty of Engineering Kuala Lumpur Malaysia; 2 Ottawa Hospital Research Institute Ottawa, ON Canada; 3 The Chancellery University of Malaysia Terengganu Malaysia

**Keywords:** box and block test, Intrinsic Motivation Inventory, maximum voluntary contraction, motor rehabilitation, upper limb amputee, video games

## Abstract

**Background:**

Brain plasticity is an important factor in prosthesis usage. This plasticity helps with brain adaptation to learn new movement and coordination patterns needed to control a prosthetic hand. It can be achieved through repetitive muscle training that is usually very exhausting and often results in considerable reduction in patient motivation. Previous studies have shown that a playful concept in rehabilitation can increase patient engagement and perseverance.

**Objective:**

This study investigated whether the inclusion of video games in the upper limb amputee rehabilitation protocol could have a beneficial impact for muscle preparation, coordination, and patient motivation among individuals who have undergone transradial upper limb amputation.

**Methods:**

Ten participants, including five amputee participants and five able-bodied participants, were enrolled in 10 1-hour sessions within a 4-week rehabilitation program. In order to investigate the effects of the rehabilitation protocol used in this study, virtual reality box and block tests and electromyography (EMG) assessments were performed. Maximum voluntary contraction was measured before, immediately after, and 2 days after interacting with four different EMG-controlled video games. Participant motivation was assessed with the Intrinsic Motivation Inventory (IMI) questionnaire and user evaluation survey.

**Results:**

Survey analysis showed that muscle strength and coordination increased at the end of training for all the participants. The results of Pearson correlation analysis indicated that there was a significant positive association between the training period and the box and block test score (*r*_8_=0.95, *P*<.001). The maximum voluntary contraction increment was high before training (6.8%) and in the follow-up session (7.1%), but was very small (2.1%) shortly after the training was conducted. The IMI assessment showed high scores for the subscales of interest, perceived competence, choice, and usefulness, but low scores for pressure and tension.

**Conclusions:**

This study demonstrated that video games enhance motivation and adherence in an upper limb amputee rehabilitation program. The use of video games could be seen as a complementary approach for physical training in upper limb amputee rehabilitation.

## Introduction

A myoelectric prosthesis is a popular choice among upper limb amputees [1], although most users stop using it after sometime owing to control difficulties, muscle fatigue, and lack of motivation to practice before getting used to the control mechanism [2]. Myoelectric prothesis task training is required before and after prosthesis fitting [3], and it takes several months of practice under the guidance of physicians and therapists to regulate these prostheses naturally. This process is exhausting and tedious [4].

The use of interactive technology can foster intrinsic motivation and thus the effort invested in the training of neuromuscular rehabilitation [5]. Various computer-based systems, including video games, have been suggested [6-9] to support motor training. Physiological data suggest that gaming can cause neuroplastic reorganizing, leading to the long-term retention and transfer of skills; however, further clinical research is needed in this field [10]. Besides, it has been shown that virtual reality (VR) platforms, in the form of video games, provide amputees with an interactive and immersive technique for enhanced muscle coordination and overall control, which has been found to be very helpful for a patient to start training before getting an actual prosthetic hand [11].

Previous studies have shown that rehabilitation tasks based on a fun and playful concept provide better outcomes compared to traditional physiotherapy exercises [12,13]. Video games have been shown to offer some positive effects on behavior and physiology [10]. Participants who learn motor skills through games, especially in the VR setting, can have better skills in the real environment [14]. Therapy needs to have high repetition, supervision, clear rewards, and a long duration over time for maximum effectiveness [15,16]. In this case, patient motivation needs to be maintained in order to increase patient engagement and the dosage of therapy-relevant movements by incorporating these movements into an interactive environment that can enhance cognitive, motor, and affective measures [10]. Video game–based rehabilitation is popular in geriatric populations [17], people with stroke [18,19], and people with Parkinson disease [20,21]. In 2018, a similar study on upper extremity amputees and able-bodied participants showed an overall improvement in electromyography (EMG) control, fine muscle activation, and electrode separation [4].

Patient motivation was usually evaluated using Intrinsic Motivation Inventory (IMI) questionnaires and observations by physiotherapists [22-24]. This study investigated whether the inclusion of video games in the upper limb amputee rehabilitation protocol could have a beneficial impact on the course of treatment and patient motivation, with the hypothesis that the functional outcome of motor rehabilitation is directly proportional to the duration of the therapeutic session [25-27] and patient interest to take part in rehabilitation [28]. Other study outcomes measured in this work are maximum voluntary contraction (MVC) readings of the forearm muscle and the box and block test (BBT) score.

## Methods

### Participants

The participants of this study were five able-bodied and five transradial amputee subjects with a mean age of 26.3 years (SD 4.47), who were recruited from University Malaya Medical Centre (UMMC), Kuala Lumpur, Malaysia. Two out of the five transradial amputee participants had congenital amputation, while the other three had undergone amputation owing to traumatic injury at a mean of 3.7 years (SD 1.69) at study inclusion. One participant was a bilateral amputee; therefore, only one residual limb was considered in this study. The rest of the amputee participants had unilateral amputation (right side for all), and the amputation level ranged from long to short transradial amputation. All amputee participants had never used any prosthetic device since amputation and had no experience with an EMG system. The amputee participants were independent, could perform all activities of daily living on their own, and received no other treatment or rehabilitation throughout the study period. In this study, the amputee participants represented the test group and the able-bodied participants represented the control group.

The EMG signal generated by each participant’s forearm was evaluated before the training session. Four video games (Crate Whacker, Race the Sun, Fruit Ninja, and Kaiju Carnage) and their respective control variations were assessed for suitability to be used in the rehabilitation protocol. All participants had normal vision and were guided throughout the study. Two questionnaires were administered (modified version of the IMI and the user evaluation study). The IMI questionnaire was administered at the end of the session, whereas the user evaluation study was administered at the end of each game on the 10th training session. The test was approved by the Medical Research Ethics Committee and the Ministry of Health Malaysia (approval ID: NMRR-16-2106-32880). The Medical Research Ethics Committee considered that the data collection for this study would only involve physical evaluation. Participants were required to sign a written consent form prior to the tests.

### Games

**Figure 1:**
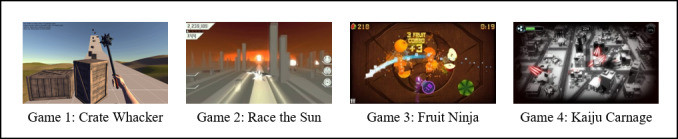
Selected open-source games.

The following four different games were used in this study: Crate Whacker, Race the Sun, Fruit Ninja, and Kaiju Carnage (Figure 1). The games were selected because of the suitability of the main player control to prosthesis usage and the compatibility with the Myo armband (Thalamic Lab). The main control used for Crate Whacker and Fruit Ninja is “make a fist,” which can improve the overall strength of the forearm muscle. The amputee participant’s residual muscle must be in good condition [29,30], and precise EMG control ability is required to control the prosthesis naturally [31]. Meanwhile, Race the Sun and Kaiju Carnage were included because of their ease of control. The game control methods are summarized in Table 1.

**Table 1:** Game control methods with the Myo armband.

Game	Player control	Action	Muscle contractions
Crate Whacker	Make a fist	Equip mace	Quick but frequent contraction
Crate Whacker	Spread the fingers	Equip grenades	Quick but frequent contraction
Crate Whacker	Wave left	Centre your arm	Quick but frequent contraction
Crate Whacker	Wave right	Reset player	Quick but frequent contraction
Crate Whacker	Pan	Smash crate	Quick but frequent contraction
Race the Sun	Make a fist	Center position	No contraction
Race the Sun	Spread the fingers	Open menu	No contraction
Race the Sun	Wave left	Mouse click	No contraction
Race the Sun	Wave right	Enable/disable mouse control	No contraction
Race the Sun	Pan	Fly side to side	No contraction
Race the Sun	Rotate	Jump up or down	No contraction
Fruit Ninja	Make a fist	Cut the fruits (hold)	Prolonged contraction
Fruit Ninja	Double tap	Move mouse	Prolonged contraction
Fruit Ninja	Pan	Unlock (hold) or lock	Prolonged contraction
Kaiju Carnage	Make a fist	Charge/release kaiju shockwave	Combination of gestures
Kaiju Carnage	Wave left	Recalibrate Myo armband	Combination of gestures
Kaiju Carnage	Pan	Quickly swipe with the hand for kaiju claw	Combination of gestures
Kaiju Carnage	Rotate Hold arm up	Charge/unleash kaiju beam	Combination of gestures

The keyboard mapper utility available in Myo Connect was used to assign keyboard and mouse commands to any of five gestures to control the game. The gestures used to control the games included fist, wave in, wave out, finger spread, and double tap. Pan motion from highly sensitive motion sensors embedded in the Myo armband is important as it allows the player to move in the game setting. The custom configuration for each game is exported as connector scripts. In Crate Whacker, the player repeatedly smashes the crate with a mace and destroys them with grenades. The player controls the mace position with the acceleration and gyroscopic data of the armband. In Race the Sun, the player controls a solar-powered spacecraft, dodging various objects on the way, such as laser beams, other ships, and other stationary obstacles, while collecting pick-ups that can be used in the game, with the sun slowly setting on the horizon. The game ends either when the player hits an object and the ship gets destroyed or when the sun sets. Meanwhile in Fruit Ninja, the player needs to slice the fruit into half to collect point. The Kaiju Carnage game enables the player to claw, smash, and shoot nuclear lasers on buildings.

### Experimental Protocol

The set up for this study includes a monitor that displays the game that the participant plays with the Myo armband connected via Bluetooth. The system utilized commercially available equipment of the Myo gesture control armband. It consists of eight plastic pods held together by a rubber lining that measures 7.5 inches (19.05 cm) in circumference and can be extended to 13 inches (33.02 cm). There are three medical steel EMG sensors on the bottom of each pod, which are responsible for reading the muscles’ electrical activity and arm movement. With the use of a machine learning process, the armband can recognize the gesture perform based on the electrical activity of the arm in real time. Amputee participants wear the Myo armband on the affected limb. Able-bodied participants wear the armband on the dominant hand. Ten 1-hour sessions were carried out within a 4-week period. Participants were initially instructed to perform the VR BBT designed by the authors, and provisional MVC levels were recorded before the four Myo-controlled computer games were introduced to each participant in a randomized order. Randomization of the games was performed by the study facilitator. At the end of the training session, the MVC value was recorded again, and a modified IMI questionnaire and a simple user evaluation survey regarding the gaming experience, similar to the approach in the study by Prahm et al [32], were administered to the participants. Follow-up MVC values were taken 2 days after the last intervention session.

### Assessment

**Figure 2:**
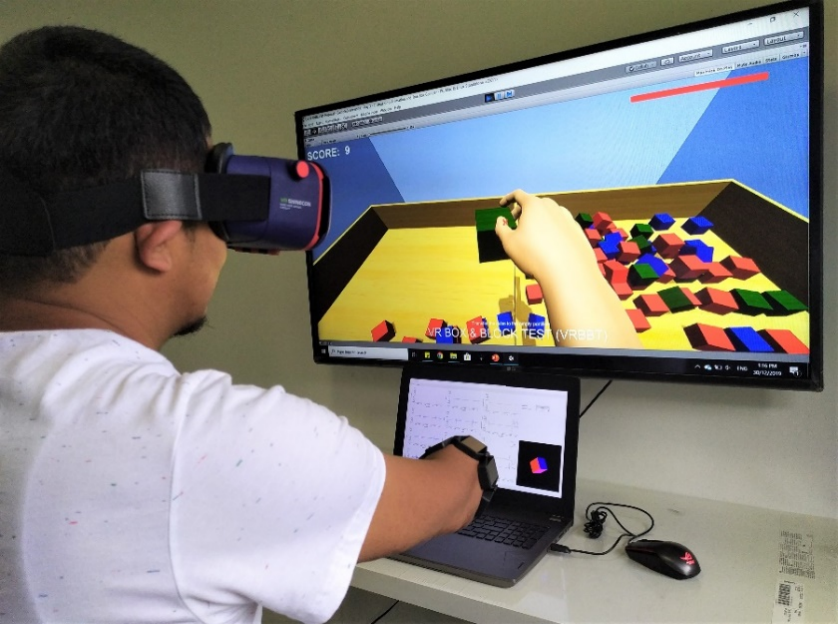
A transradial amputee participant performing the virtual box and block test.

In order to investigate the effect of the video game rehabilitation protocol used in this study, VR BBTs and basic EMG assessments evaluating approximate coordination and muscle strength were performed. The participants were asked to perform the BBT in a virtual environment (Figure 2). The test was made up of the following two phases: an initial 15-second phase, where the user gets a trial run, followed by a scored 60-second phase. Participants were told to move as many blocks as possible in a span of 60 seconds (one by one) from one compartment to another. The participant’s hand must cross over the partition in order for a point to be given, and blocks that drop or bounce out of the second compartment onto the floor are still rewarded with a point. The instructions are scripted, and they were read to each user according to the manual for the test. The experiments were performed according to the World Medical Association Declaration of Helsinki [33]. The player needs to move the virtual hand near the cube, contract the forearm muscle to pick the cube up, and then stop contracting the muscle to release the cube into another compartment. The score generated on the top left of the screen refers to the amount of successfully transferred blocks, and the red line on the top right corner of the screen refers to the linear timer (Figure 2).

**Figure 3:**
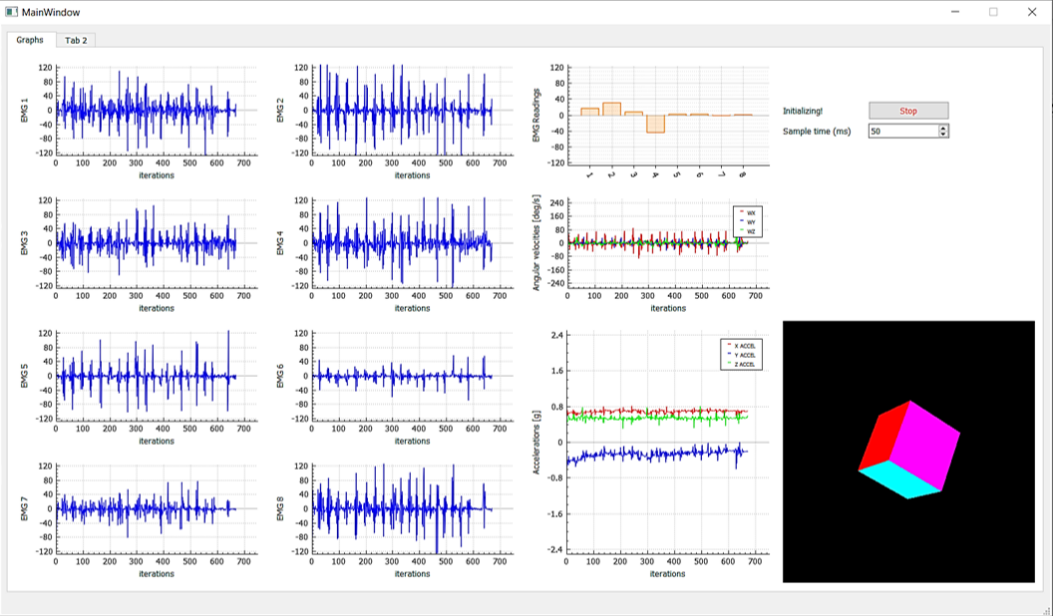
The Myo visualization tool interface that streams the electromyography (EMG) signal detected by the armband. The first two columns represent the EMG sensors detected by pods 1-8. In the third column, the first graph on the top is the EMG representation as a whole, while the other two are angular velocities and accelerations.

The MVC was recorded for each participant before, immediately after, and two days after playing the game. Participants were instructed to maximally contract their forearm muscle and to hold this contraction for 10 seconds, and the highest value was recorded as the MVC value. The assessment was carried out with the Myo visualization tool (Figure 3) on a laptop with a discrete graphics processing unit (NVDIA GeForce GTX 1060) for signal acquisition, filtering, calibration, training, and prediction. The Myo visualization tool is an open-source Myo armband data reader created using Myo SDK, Qt Creator, and QCustomPlot that streams and displays the EMG signal and accelerometer, gyroscope, and orientation data of the Myo armband.

### Questionnaires

Two questionnaires were provided for each participant to be completed at the end of the training session, which included a modified IMI questionnaire [3] and a user evaluation survey [32]. The participants’ experiences with each game were evaluated using the IMI questionnaire, which has previously been used with a virtual environment for motor rehabilitation [22-24]. A modified version of the IMI questionnaire made up of five selected subscales was utilized to evaluate participants’ experiences with the protocol. The subscales included were interest/enjoyment, perceived competence, perceived choice, pressure/tension, and value/usefulness. The questionnaire included statements such as “I found this activity very interesting,” and they were rated on a 7-point Likert rating scale from 1 (not true) to 7 (true).

A brief questionnaire about the gaming experience was presented after the completion of each game. This short survey included questions about the gameplay, fun factor, motivation, and input and control methods, and it included the following questions: (1) Did you like this game in general? (2) Did you have fun playing the game? (3) How would you rate the input mechanism? (4) How would you rate the game control? (5) Did this game motivate you? Question could be answered on a 5-point Likert scale ranging from 1 (“do not agree”) to 5 (“agree”). The participants were asked to focus on the differences between each game and to avoid giving the same answer to one statement for all the games if possible.

## Results

In this study, we showed that incorporating video games in an upper limb amputee rehabilitation protocol would provide benefits to the rehabilitation output. The platform consists of four video games controlled by an off-the-shelf Myo gesture control armband. The games were played by five able-bodied and five transradial amputee participants. The use of the four video games was widely explored in this context. No technical difficulties were encountered with the game settings.

**Figure 4:**
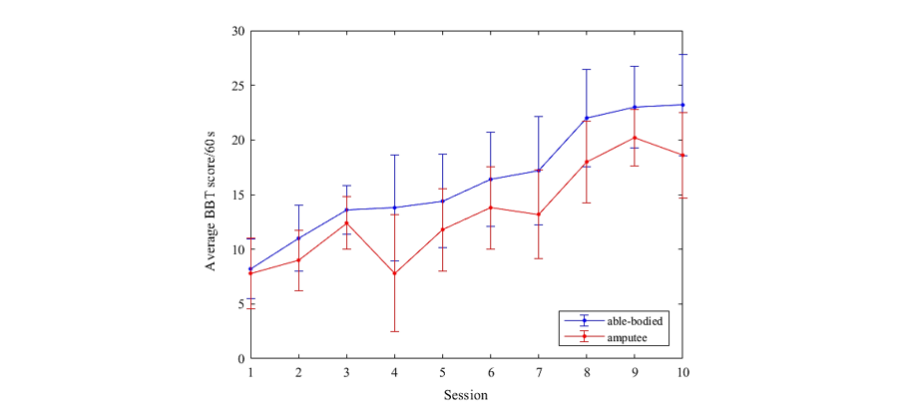
Mean score in the box and block test per 60 seconds over 10 training sessions.

The average BBT score in every training session gradually increased, with the mean score reported in the 10th training session of this study being 23.2 (SD 4.62) in the control group and 18.6 (SD 3.93) in the test group (Figure 4). The results of the Pearson correlation assessment indicated that there was a significant positive association between the training period and the BBT score (*r*_8_=0.95, *P*<.001). The highest score achieved was 29 blocks transferred over 60 seconds by one able-bodied participant during the final session.

**Figure 5:**
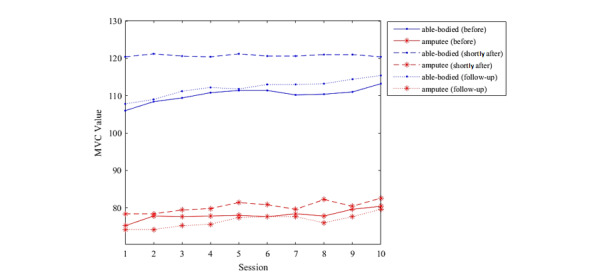
Mean maximum voluntary contraction (MVC) values before and shortly after training and during the follow-up session.

From sessions 1 to 10, the MVC value for able-bodied participants increased during follow-up and shortly after the training was conducted, but not immediately after the training session. This may be caused by muscle fatigue. Meanwhile, for amputee participants, the MVC value increased before and shortly after the training was conducted, and during the follow-up session (Figure 5). This proves that the muscle strength of all participants increased with the training duration. Shortly after the training session, six out of 10 participants showed an improvement in the MVC value from the initial to the final session, two participants showed no improvement, and two participants showed a reduction in the MVC value at the end of the training session. In the follow-up session, nine out of 10 participants showed an improvement in the MVC value compared to the initial training session. The MVC increment was greater before training and in the follow-up session but was very small (approximately 2.1%) shortly after the training was conducted.

The results obtained from the IMI questionnaire are shown in Table 2. Participants had high interest in playing the games, perceived playing the games as their own choice, and felt competent and at ease while playing the games. Participants also agreed that this activity provided benefits that included strengthening their muscles, motivating them, and keeping them active, resulting in higher motivation to perform the activity for a longer period of time. Pressure or tension was the only subscale with a low reported average score. The difference in the scores for both groups was insignificant for all subscales, except for the perceived choice subscale (*d*=0.58).

**Table 2:** Intrinsic Motivation Inventory questionnaire results based on five subscales.

Subscale	Likert scale score, mean (SD)^a^	
	Able-bodied participants	Amputee participants
Interest/enjoyment	6.8 (0.40)	6.6 (0.49)
Perceived competence	6.2 (0.75)	6.2 (0.40)
Perceived choice	5.8 (0.75)	4.4 (0.49)
Pressure/tension	1.8 (0.75)	2.0 (0.63)
Value/usefulness	6.4 (0.80)	6.2 (0.40)

^a^Score range: 1 (low) to 7 (high).

According to the results of the user evaluation survey (Table 3), the favorite game (derived from the score of question 1 and question 2) was Race the Sun, followed by Fruit Ninja, Crate Whacker, and Kaiju Carnage. There was no difference in the distribution of the games for both general and fun categories, but a significant difference was observed in Crate Whacker, where amputee participants appeared to less enjoy Crate Whacker compared with able-bodied participants (*P*<.001).

**Table 3:** Results of the user evaluation survey.

Category	Score							
	Able-bodied participants				Amputee participants			
	Crate Whacker	Race the Sun	Fruit Ninja	Kaiju Carnage	Crate Whacker	Race the Sun	Fruit Ninja	Kaiju Carnage
General	3.6	4.2	4.2	1.6	3.2	4.4	4.2	1.4
Fun	3.6	4.2	4.2	1.6	3.0	4.2	3.8	1.6
Input	3.6	3.8	4.0	1.6	3.4	4.2	4.4	1.4
Game control	4.0	3.8	4.2	1.8	3.0	4.2	4.6	1.2
Motivation	3.8	3.8	4.4	1.6	3.6	3.8	4.0	1.6

## Discussion

### Principal Findings

An important finding from this study lies in the usage of the platform in 10 1-hour sessions within a 4-week period. The BBT is a standard test to assess hand functionality [34,35]. In this study, the test was used in a virtual setup in order to check the participants’ ability to control and operate a myoelectric hand, as the mechanism utilized to pick and place an object in the virtual setting is similar to that for a myoelectric prosthesis. Compared to the mean conventional BBT score for a healthy person as reported by Mathiowetz [34], which is in the range of 78.9 (SD 15.3) to 75.1 (SD 11.1) per 60 seconds, the score for the virtual setup was very low (approximately 74% lower score). However, the mean score increased from session 1 to 10.

The MVC test is a standardized method for measurement of muscle strength [36]. The results obtained demonstrate improvements in muscle strength from pregaming to follow-up assessments. A high increment was observed among amputee participants after playing the game, which is consistent with the finding in the study by Prahm et al [32]. This result may be associated with the electrode resistance that may be affected by a change in body temperature or sweat, which, at the same time, proves that the use of video games in upper limb amputee rehabilitation is not fatiguing for the participants. It can be safely said that certain improvements in EMG control could be detected by the MVC test. Besides, from the results, MVC values recorded for able-bodied participants were greater than those for transradial amputee participants, with the lowest MVC value recorded for an amputee participant with congenital amputation, which may have been associated with muscle atrophy. Atrophy of the remaining muscle occurs when the muscle is not used for long periods of time, and the EMG signal becomes very weak after amputation [37]. Male participants recorded greater MVC values compared to female participants.

Based on the IMI questionnaire and user evaluation survey conducted, the motivational aspects of training gamification were traceable. Participants agreed that the activity can induce desirable physiological changes that are consistent with the findings in the study by Lohse et al [10]. Pressure (psychologically) could be felt by amputee participants while playing the Race the Sun game because of its difficulty level. Nevertheless, the participants were still motivated to play the game according to the survey conducted. It has been reported that enjoyment, control mechanisms, music, direct feedback on scores, and ability to receive upgrades influence a player’s motivation [32], and choice, rewards, and goals lead to increased engagement [10].

Aside from game suitability and main player control, the four games were included in this study because each of the games involved a different engaging element. Crate Whacker is continuous without a specific time limit, scoring system, and level upgrade. Race the Sun and Fruit Ninja have both a scoring system and specific time to complete the game, but the player can receive upgrades only in Race the Sun. Meanwhile, in Kaiju Carnage, a specific time limit is available, but the player receives no score or upgrade. All games, except for Kaiju Carnage, have no background music. The majority of the participants reported that they enjoyed and wanted to continue playing Race the Sun and Fruit Ninja after the training period, which indicated increased participant motivation as reported in previous studies [25-27]. Both of these games provide direct feedback for the player interaction, and the player can see the timer and score obtained clearly while playing, which is supported by the findings of Liepert et al [15]. According to question 3 and question 4, participants preferred the control in Fruit Ninja, as they felt that they were slicing the fruit in the game with their own hands. A significant difference in the score distribution was observed in Crate Whacker and Kaiju Carnage under game control input (*P*<.001), indicating that amputee participants had control difficulty in these games. In terms of motivation (question 5), all of the participants only felt less motivated while playing Kaiju Carnage owing to lagging in EMG control and long loading time. Participants also reported that this game showed no score while playing, had a low level of difficulty, and was not interesting. In addition, all amputee participants appeared to be struggling to imitate the clawing gesture, but this was not the case with able-bodied participants. This part of the study showed that engaging elements contribute to increasing a player’s motivation during rehabilitation.

### Conclusions

This study demonstrated that a video game–based rehabilitation protocol can be used as a complementary method for upper limb amputee rehabilitation. Participants showed greatly improved muscle strength, coordination, and control. A good muscle condition and induced neuroplasticity enabled better control in the BBT, which is related to readiness to use a myoelectric prosthesis. Participants agreed that this activity is beneficial to them both behaviorally and psychologically. According to the questionnaire responses, it could be shown that the use of video games maintains patient interest during training, therefore improving patient motivation and exercise intensity and enhancing adherence throughout the rehabilitation period. The engaging elements in the game could be identified with the questionnaire. Video games with high intensity and difficulty create pressure for the player, but not all Myo-controlled video games are suitable for transradial amputee rehabilitation.

## Abbreviations

BBT: box and block test
EMG: electromyography
IMI: Intrinsic Motivation Inventory
MVC: maximum voluntary contraction
VR: virtual reality
